# Use of physical activity skills in daily life during a physical activity intervention for women in midlife with elevated risk for cardiovascular disease

**DOI:** 10.1007/s10865-026-00665-3

**Published:** 2026-04-20

**Authors:** Gabrielle M. Salvatore, Iris Bercovitz, Angelica R. Rivera, Giada Benasi, Emmanuel Lapitan, Amanda L. Folk, Kyle R. Haggerty, Jacqueline A. Mogle, Danielle Arigo

**Affiliations:** 1 Department of Psychology, Rowan University, 201 Mullica Hill Road, Robinson Hall 116G, Glassboro, NJ 08028 USA; 2 Hunter Holmes McGuire VA Medical Center, Richmond, VA USA; 3 RTI Health Solutions, Durham, NC USA; 4 Department of Family Medicine, Rowan-Virtua School of Osteopathic Medicine, Stratford, NJ USA

**Keywords:** Psychological skills, Behavioral intervention, Daily diary, Women’s health, Midlife, Physical activity

## Abstract

Women in midlife (ages 40–65) often fail to engage in cardioprotective levels of physical activity (PA). Although behavioral PA interventions are tailored for this population, the extent to which women use the skills introduced by these interventions in daily life is unclear. In the present single-arm trial, 62 women in midlife with ≥ 1 CVD risk factors (e.g., hypertension; *M*_*Age*_ = 52, *M*_*BMI*_ = 31 kg/m^2^, 40% racial/ethnic minority identification) engaged in an 8-week PA program that taught 9 distinct behavioral, cognitive, and acceptance-based PA skills. Participants wore a PA monitor each day to capture their steps per day and daily skill use was assessed with an end-of-day survey; survey compliance was high (84%), and participants reported using an average of 2.2 skills per day. Within-person, use of an additional skill per day corresponded to 550 more steps per day (*p* < 0.001). Use of some PA skills changed over time and the number of skills used was slightly lower on days with intervention sessions than days without (*F*[1,61] = 3.99, *p* = 0.05). End-of-treatment ratings showed that reporting on skill use each day was highly acceptable (*M* = 4/5). Findings show that participants can report on their use of PA skills between sessions and use of these skills in daily life is associated with the intended behavior change and subjective experience. Additional examination of associations between daily skill use and behavior change will enable improvements to intervention programs, to better engage or remove lesser-used skills as appropriate.

## Introduction

Interventions to promote health behavior change facilitate the acquisition of new psychological and behavioral skills, either via training with an interventionist or through a digital system. Skills training draws on evidence-based intervention approaches such as Cognitive-Behavioral Therapy (CBT), Acceptance and Commitment Therapy (ACT), and motivational interviewing (Vidic & Cherup, 2022) and often focuses on a discrete set of behavior change techniques (BCTs; Marques et al., 2021), to harness specific mechanisms of action that are associated with health behavior change (cf. Arigo et al., 2022). For example, self-monitoring provides feedback on progress and can prompt self-efficacy for continuing change efforts, which promotes the achievement of health behavior goals (Strecher et al., 1986). The ultimate goal of psychological skills training is for participants to apply the skills taught in their daily lives, to overcome barriers to healthy behaviors and build sustainable habits (Ramji et al., 2020).

Consequently, the success of a behavior change program depends largely on participants’ application of the skills they learned during intervention sessions to their daily lives. Yet, little is known about participants’ use of the skills taught in health behavior change interventions in their natural environments, between intervention exposures, including behavior change interventions for physical activity (PA). PA is critical to the prevention and mitigation of chronic conditions (e.g., cardiovascular disease, type 2 diabetes; Anderson & Durstine, 2019; Kaminsky et al., 2022) and also contributes to improved mental health, reduced healthcare costs, and increased quality of life (Anderson & Durstine, 2019). Inclusion of specific skills in interventions is associated with improvements in PA outcomes, such as goal setting and problem-solving (Carraça et al., 2021), though there is little evidence to indicate the extent to which these skills are applied in daily life. Investigation of participants’ PA skill use in their daily lives, between skills training sessions, would help to identify critical missing links in our understanding of mechanistic pathways as well as changes in skill use over time. This work could also identify gaps in the translation of skill acquisition to regular practice, thereby enabling improvements to the design and implementation of PA behavior change programs.

Intensive assessment methods involve participants reporting on the extent to which they experience particular events, emotions, and behaviors as they go about their normal activities (cf. Lischetzke & Könen, 2021; Schneider & Stone, 2016). This approach has many advantages over traditional global, retrospective self-report: it increases accuracy and precision of self-reports by limiting recall bias and offers insight into how processes may differ *for the same person across days* (Smyth et al., 2017). Daily self-reports can also be linked with ambulatory, device-based behaviors (e.g., PA; Arigo et al., 2020). With respect to psychological skills used (or not) between intervention sessions, daily assessment of PA skill use would provide a clear and nuanced picture of which skills are used in daily life and whether their use is associated with behavior change over the course of an intervention (cf. Southward et al., 2022). Such information would allow for evaluating which skills are used more (vs. less) often and which are most strongly associated with behavior change, informing improvements to interventions (e.g., removing lesser-used skills or revising how these skills are taught, to increase ease of implementation in daily life).

Further, as noted, PA skills are intended to help individuals overcome barriers and build sustainable habits (Ramji et al., 2020). However, little research has explored whether barriers to PA vary or remain stable from day to day, for the same person (Dunton, 2017). Only one study has assessed PA barriers in daily life (among bariatric surgery patients), and the variability in barriers experienced was not reported (Bond et al., 2013). Although PA skills are usually designed to address specific barriers rather than all potential obstacles, it is not clear whether skills are indeed used in response to specific barriers; intensive assessment methods offer a valuable opportunity to explore this question.

### Approach and aims of the present study

As part of a long-term program of research, a PA skills training intervention was tailored to meet the needs of insufficiently active women in midlife (ages 40–65) with elevated risk for cardiovascular disease (CVD). During midlife, biological aging and the onset of menopause sharply increase women’s risk for CVD (Matthews et al., 2009), and this risk is compounded by decreases in PA (which does not occur among men; Lee, 2005). Many interventions already exist that purport to be tailored for this large, heterogeneous, and high-risk population (Arigo et al., 2022). As they are only minimally effective (Murray et al., 2017), however, there is a clear need for improvements that can help to overcome this group’s particular barriers (e.g., limited time, heightened stress, unpredictable schedules due to caregiving burden, all-or-nothing thinking that limits small changes when larger ones are not possible; Cadmus- Bertram et al., 2020; Adachi-Mejia et al., 2010; Satish et al., in press; Tudor-Locke et al., 2003).

The present analyses were selected to examine (1) the feasibility and acceptability of daily assessment of psychological and behavioral skill use during an 8-week PA promotion intervention, (2) the extent to which participants reported using different skills taught in their daily lives, (3) the day-level association between PA barriers and skills used in daily life, and (4) the day-level associations between skills used and PA behavior, as well as between PA barriers and PA behavior, over the course of the intervention. We hypothesized that the use of PA skills would be highly variable within-person (reflecting low stability across days), that the number of skills used would be positively associated with PA behavior at the day level, and that the likelihood of reporting skill use would be higher on days when women did (vs. did not) experience barriers to engaging in PA. All other tests were conducted in an exploratory manner.

## Methods

### Recruitment and participants

Women were recruited between January and May 2024 via social media advertisements, announcements to the campus community at the supporting institution and in associated family medicine clinics, and from an existing database of previous study participants. Those eligible were women ages 40–65 (inclusive) with one or more CVD risk conditions (i.e., hypertension or prehypertension, type 2 diabetes or prediabetes, metabolic syndrome, current smoker, quit smoking in the past 3 months, high cholesterol) who engaged in < 90 min of moderate-to-vigorous PA per week or < 7000 steps per day prior to the program. Eligibility also required fluency in English, not pregnant, no medical or psychological limitations that could interfere with participation (e.g., injury, psychosis), and consistent internet access via a personal device. The final sample included 62 women (*M*_*Age*_ = 52, *M*_*BMI*_ = 31 kg/m^2^): 40% identified with a racial/ethnic minority group and the largest subsets indicated high socioeconomic status (41.9% reported having a graduate degree; 81.3% indicated an annual household income > $50,000). Additional demographic information can be found in Table 1. All participants were retained through the end of the program (100%); 97% of intervention sessions were completed; 92% of participants completed all sessions (*n* = 57) and 8.0% (*n* = 5) missed 1–2 sessions due to unexpected circumstances (e.g., medical or caregiving needs). A subset of participants were also invited to complete an exploratory 3-month follow-up week of observation.

**Table 1:** Participant demographics (*N* = 62)

	*M (SD)*, Range
Age (years)	52.6 (6.7), 41-65
BMI (kg/m^2^)	32.6 (7.3), 18.9-52.2
Number of CVD Risk Factors	1.6 (0.7), 1-4

	*n* (%)		*n* (%)
*Racial identification*		*Menopause status*	
Black or African American	16 (25.8)	Premenopause	14 (22.6)
White	41 (66.1)	Perimenopause	8 (12.9)
Native Hawaiian or Other Pacific Islander	1 (1.6)	Postmenopause	23 (37.1)
Other	4 (6.4)	Other	17 (27.4)
*Ethnic identification*		*Children*	
Hispanic or Latina	8 (12.9)	No	12 (19.4)
Non-Hispanic or Latina	54 (87.1)	Yes	50 (80.6)
*Highest education level*		*CVD risk conditions*	
High school graduate/GED	2 (3.2)	Hypertension/prehypertension	32 (51.6)
Associate’s degree, partial college, or technical degree	11 (17.7)	Type 2 Diabetes/prediabetes	16 (25.8)
Bachelor’s degree	23 (37.1)	Metabolic Syndrome	4 (6.4)
Graduate degree	26 (41.9)	Current smoker	4 (6.4)
		Quit smoking in past 3 months	2 (3.2)
		High cholesterol	42 (67.7)
*Marital status*		Household income*	
Married	35 (56.4)	<$25,000	3 (5.1)
Widowed	4 (6.4)	$25,000 to $50,000	8 (13.6)
Divorced	9 (14.5)	$50,000 to $75,000	15 (25.4)
Separated	3 (4.8)	>$75,000	33 (55.9)
Single	10 (16.1)		
Other	1 (1.6)		
*Provide childcare*		*Provide other care*	
No	56 (90.3)	No	54 (87.1)
Yes	6 (9.7)	Yes	8 (12.9)

^*^n = 59

### PA intervention philosophy

The present study built on preliminary work (Arigo et al., 2025) to provide an 8-week PA intervention that emphasized small, sustainable changes to PA and a flexible approach. The focus was on *lifestyle PA,* or overall movement at any intensity for any duration, rather than structured bouts of exercise (e.g., taking the stairs instead of the elevator; Heesch et al., 2003; Dąbrowska et al., 2015; Dearden & Sheahan, 2002; Jurakićć, Pediššić & Greblo, 2010). As such, the primary PA outcome of interest was steps per day, representing overall movement at any intensity. As women in midlife often cite lack of social support for PA as a barrier to engagement (McArthur et al., 2014), the intervention also paired participants in partner dyads to bolster PA-specific social support. Partners were paired based on age, starting level of PA, and mutual availability for intervention sessions. Partners were encouraged to communicate with each other to provide peer support between intervention sessions.

PA skills coaches who delivered the intervention were behavioral science trainees with experience in women’s health and PA promotion and taught participants a combination of behavioral, cognitive, and acceptance-based skills. Please see Table 2 for a description of each PA skill introduced in the program and the timing of its introduction. During each coaching session, the coach provided psychoeducation, checked in on PA progress, and helped participants self-select weekly PA goals. Rather than applying a standardized protocol or prescription, coaches adopted an individualized approach: goals were selected based on participants' preferences and baseline PA levels and operationalized in two categories: lifestyle or overall PA goals (in steps per day) and exercise goals (in minutes of moderate-to-vigorous intensity activity). Participants were encouraged to set overall PA goals and had the option to set exercise goals. There was no algorithm or upper limit for progressive goals; as our program was entirely personalized, goals were tied to participants' starting levels of PA and their progress each week.

**Table 2:** Description of physical activity promotion skills introduced in the 8-week intervention

Skill	Description	Week introduced
Goal setting	Using a goal sheet and activity planner to plan out the week and as reminders	2
Time management	Making PA a “big rock” and building other responsibilities around it	2
Values	Keeping values in forefront of mind; “voting up” for own values	2
Willingness	Engaging in a valued behavior regardless of thoughts/feelings (e.g. “even if”/”only if”)	2
Partner support	Making changes by connecting with partner (e.g., accountability, brainstorming), communicating needs and responding to partner’s needs	2
Lapse vs. relapse (and other negative self-talk)	Remembering that increasing PA is a process that will involve slips (or lapses); a lapse is not a relapse, ask yourself key questions and make a plan to start now; look to your partner (and coach) for support	3
Problem-solving	Taking a few minutes to reflect on what’s working/not working, brainstorming, talking to partner, making a plan to create new links	4
Cognitive defusion	Choosing actions independent from immediate thoughts and feelings	5

### Procedure

This study was approved by the (Rowan University) Institutional Review Board (No. PRO-2023-355; clinical trials registration NCT06350604) and all participants provided written documentation of informed consent. Interested individuals completed a brief survey and were scheduled for a subsequent eligibility screening call. Those who were eligible and remained interested were introduced to study procedures and provided their availability for the first intervention session; they completed a baseline survey and 7 days of observation prior to the first session (Week 1). During this observation period, participants were asked to wear a PA monitor each day (i.e., study-issued pedometer or personal PA monitor) and complete an end-of-day survey within an hour of going to bed. As described in detail below, the end-of-day survey assessed the primary barrier to PA that day, any PA skills used to support their PA, and PA behavior (recorded in steps per day). Participants reported their steps on the end-of-day survey by reading and copying their step count from their PA monitor. These values were reviewed by PA coaches and trained research staff and used to support goal selection.

Participants were paired with a PA partner and dyads were assigned a PA coach at the end of Week 1, before they started the 6 weeks of active intervention for PA skill building (Weeks 2–7). Weekly sessions included three 90-min remote meetings via Zoom with PA partners and their coach for skill building (Weeks 2, 5, and 7) and three 20-min individual phone calls with their coach for additional skill building and professional support (Weeks 3, 4, and 6). Participants continued to complete daily end-of-day surveys for the remainder of the program, with the inclusion of a survey item to assess the implementation of PA skills used that day. The 6-week intervention was then followed by a final week of post-intervention observation (Week 8). During this time participants continued daily self-monitoring and partner communication but did not receive coaching. At the end of the program, all participants completed a survey of program acceptability.

A follow-up week of assessment was added to the protocol during the study, after some participants had already completed the program and returned their PA monitors. Those who were still enrolled or who still had access to their monitors were invited to complete a 7-day follow-up assessment at 12 weeks post-intervention, which included only PA monitor wear and end-of-day surveys. For those who participated, communication with their assigned PA partner was not required but was assessed for maintenance of contact. Of those invited (*n* = 54), 35 completed (65%). Participants received up to $165 as compensation for all study-related activities via an electronic debit card.

### Measures

#### Demographics

An electronic survey assessed demographic characteristics including race and ethnicity, age, menopause status, marital status, caregiving status, income, education, height, and weight. Height and weight were used to calculate body mass index (BMI).

#### Daily reports of skill use and barriers to PA

Participants completed a survey at the end of each day that was intended to take ≤ 5 min. They were asked to report on their daily use of PA skills, whether they communicated with their PA partner that day, and daily PA barriers. Survey items (e.g., “*Which physical activity skills (if any) did you use to be active today?*”) were based on previous intensive assessment studies (Dockray et al., 2010; Littlewood et al., 2019; May et al., 2018). See Table 3 for the daily survey items and response options.

**Table 3:** Survey questions and response options relevant to daily physical activity skill use and reporting

End-of-day survey item	Response options			
“If you encountered any barriers to physical activity today, what was your primary barrier?”	None or not applicable Fatigue Lack of time Lack of support from others	Low motivation Pain Negative self-talk Other (please describe)		
“Which of the PA skills (if any) did you use to be active today? Please check any and all that you used today.”	Other Willingness (only if / even if) Time management Communication Problem-solving	Partner support Effective goal setting Defusion from thoughts or emotions Lapse vs. relapse or other self-talk Values (such as voting up and down)		
“Which physical activity skill(s) did you discuss with your partner today?	Other Willingness (only if / even if) Time management Communication Problem-solving Partner support	Effective goal setting Defusion from thoughts or emotions Lapse vs. relapse or other self-talk Values (such as voting up and down) None		

Intervention acceptability survey item	Response options		M (SD)	Range
“How much did you like or dislike Reflecting on your day in evening surveys?”	Strongly dislike Dislike	Neutral Like	4.05 (0.86)	2.00–5.00
“How much effort did it take to engage with the requirements of the WHADE Partner Program?”	No effort at all A little effort	Neutral A lot of effort	3.14 (1.05)	1.00–5.00
“I am confident in my ability to use the skills I learned in the program, such as willingness, de-fusion, problem-solving, partner support, etc.”	Strongly disagree Disagree	Neutral Agree	4.40 (0.69)	3.00–5.00

#### PA behavior (steps per day)

PA was assessed with either a study-issued Accusplit AX2720MV pedometer (*n* = 32; 52%) or participants’ personal device (e.g., Fitbit, Apple Watch, Garmin, Samsung; *n* = 30; 48%). These devices have demonstrated reliability and validity for step tracking (Fuller et al., 2020). As reported elsewhere (Arigo et al., 2025), there was no difference between those who used a study-issued monitor versus those who used their personal PA device (*t*[61] = 0.77, *p* = 0.55).

#### Acceptability

Intervention acceptability was measured using the Theoretical Framework of Acceptability Questionnaire (TFA; Sekhon et al., 2022). In addition to general acceptability, three tailored items were relevant to participants engaging with, use of, and reporting of PA skills. The first was “*How much effort did it take to engage with the requirements of the WHADE Partner Program?*” with response options ranging from *No effort at all* (1) to *Huge effort* (5). Second, participants were prompted to respond *Strongly disagree* (1) to *Strongly agree* (5) to the following question: “*I am confident in my ability to use the skills I learned in the program, such as willingness, defusion, problem-solving, partner support, *etc*.*” Finally, participants responded to “*How much did you like or dislike the following aspects of the program? Reflecting on your day in evening surveys*.” These items were rated on a 5-point scale (*Strongly dislike* to *Strongly like*), with higher scores reflecting more favorable responses. Internal consistency across the sample was high, as indicated by Cronbach's alpha of 0.81. Please see (Arigo et al., 2025) for a full description of intervention acceptability.

### Data analysis

All analyses were conducted in SAS Version 9.4 (Cary, NC). Focusing on Weeks 2–8 only in initial analyses (i.e., during the intervention and the immediate post-intervention assessment), participants completed 2,459 end-of-day surveys of the expected 2,600 (95%); 2,186 were completed on time and were included in the present analyses (84%), affording ample power for the inferential tests described below (Maas & Hox, 2005; Murayama et al., 2022). With respect to feasibility, we set a threshold of 80% survey completion as evidence that daily assessment of skills used is feasible to implement with the population of interest, and a threshold of 4.0/5 for ratings of acceptability.

We first used descriptive statistics to understand compliance with the daily reporting protocol specific to reports of skill use and the frequencies for reporting use of each skill in daily life. Each skill was treated dichotomously for each daily report, where 1 indicated that the skill was reported as used that day and 0 indicated that the skill was not reported as used that day. We also calculated day-level totals for the number of skills used each day and the average number of skills used per person each day; these were treated as continuous for descriptive statistics (estimates and standard errors). For skills introduced later in the intervention (see Table 2), only responses recorded on days after the introduction of the relevant skill were included in descriptive statistics; percentages were calculated relative to the number of days skills were “available” throughout the intervention. We took a similar approach to describe participants’ primary barrier to PA, which they reported at the day level; this variable was treated categorically, with the 8 options listed in Table 3. As “no barriers” was the most frequent response, we also created a dichotomous variable representing a daily report of experiencing any barriers to PA (1) or endorsing no barriers to PA that day (0). We examined the frequency of reporting use of each skill taught at the day level, the range and combinations of skills used each day, and the co-occurrence of PA barriers with skills implemented, as well as change in each of these reports over time. We also evaluated person-level stability (vs. variability) in skill use and tested day-level associations between skill use, PA behavior, and PA barriers.

We then used an empty 2-level multilevel model with days nested in participants to estimate between-person stability versus within-person variability in the use of skills and the occurrence of any PA barriers (yes/no), using intraclass correlation coefficients (*ICC*s). Subsequent 2-level multilevel logistic regression models tested for change in the odds of experiencing PA barriers (yes/no) and of using each skill over the course of the intervention, which employed PROC GLIMMIX with week of assessment (time) treated continuously. Additional multilevel models tested for (1) differences in the odds of reporting barriers between days with and without intervention session, and (2) change in the number of skills used each day over the course of the intervention. In the latter models, the number of skills per day was treated continuously in PROC MIXED with restricted maximum likelihood estimation methods. Effect sizes are expressed as odds ratios or as semipartial correlation coefficients (*sr*s), respectively, and the corresponding difference or change in steps per day is reported where appropriate for additional context.

To determine the co-occurrence of specific skills at the day level, we conducted multilevel configural frequency analysis (Stemmler, 2020), which identifies high- and low-frequency patterns in binary datasets – i.e., those that occur more (“types”) or less (“anti-types”) frequently than expected. We conducted these analyses separately for days with versus without barriers to PA, for comparison. We also employed 2-level multilevel models to test for within-person associations between daily PA behavior (in steps, treated as a continuous outcome) and the total number of skills used per day (0–8, continuous predictor). Models that used these day-level (within-person) predictors also controlled for stability in these characteristics using person-level sums or means for each variable, as appropriate. Finally, we used descriptive statistics during the week of follow-up assessment to explore the sustainability of skill use.

## Results

### Feasibility and acceptability of daily skill use reporting

As noted, 2,186 out of the expected 2,600 end-of-day surveys were completed on time during the intervention (84%), meeting our specified threshold for feasibility (i.e., 80% completion). The overall mean rating of acceptability for reporting on daily use of PA skills was 4.0/5. Participants rated the effort required to complete program requirements such as daily surveys at 3.1/5 (where lower indicates less effort) and confidence in their ability to use PA skills learned in the program as 4.5/5 (see Table 3).

### Frequencies and variability in skills used

*ICC*s showed that ≤ 56% of the variability in whether a skill was reported as used each day (vs. not) was attributable to between-person stability across days, whereas 44–67% of the variability was attributable to within-person fluctuation (and error; see Table 4). Use of engaging values and problem solving were most stable between-person and use of willingness and “other” skills were least stable. “Other” skills reported included small changes (*n* = 23 days; e.g., parking farther away, increasing walking while completing daily chores), support from other people in their lives (*n* = 8 days; i.e., coaches, friends, or romantic partners), and grit or self-motivation (*n* = 3 days). The small changes listed were consistent with our program philosophy, as was engaging support from others besides their partner; the latter most closely aligns with our communication skill. In contrast, grit and self-motivation were not explicitly discussed in the program.

**Table 4:** Daily physical activity skill reported use during the intervention and immediate post-intervention weeks of reporting

	*ICC*s	Day level frequency *n* (%)	Odds of reporting use across days [95% CI]	Person level *M* number of days [range]	Person level *M*% of days [range]	Change in odds of reporting use across intervention [95% CI]
Goal setting	0.38	84 (3.9%)	0.02 [0.01, 0.04]	1.4 [0–12]	3.3% [0–30.0%]	0.82 [0.55, 1.24]
Time management	0.41	656 (30.7%)	0.25 [0.18, 0.36]	10.6 [0–32]	25.4% [0–78.0%]	1.06 [1.0, 1.13]
Values	0.56	360 (16.9%)	0.07 [0.04, 0.12]	5.8 [0–30]	13.9% [0–73.2%]	1.06 [0.98, 1.15]
Willingness	0.33	945 (44.3%)	0.43 [0.35, 0.51]	15.2 [0–37]	36.4% [0–90.2%]	1.11 [1.05, 1.18]
Partner support	0.34	640 (30.0%)	0.26 [0.19, 0.33]	10.3 [0–32]	24.9% [0–80.0%]	1.00 [0.94, 1.06]
Communication	0.38	175 (8.2%)	0.04 [0.03, 0.07]	2.8 [0–24]	6.8% [0–58.5%]	0.88 [0.79, 0.97]
Lapse vs. relapse (negative self-talk)*	0.48	146 (11.8%)	0.03 [0.02, 0.05]	2.4 [0–22]	12.3% [0–100.0%]	1.27 [1.14, 1.42]
Problem solving*	0.50	278 (20.1%)	0.10 [0.06, 0.17]	4.5 [0–25]	16.2% [0–92.6%]	0.98 [0.76, 1.26]
Defusion*	0.47	129 (12.7%)	0.06 [0.04, 0.10]	2.1 [0–12]	10.0% [0–57.1%]	1.07 [0.96, 1.20]
Other	0.33	195 (9.1%)	0.06 [0.04, 0.09]	3.1 [0–20]	7.5% [0–48.8%]	1.35 [1.23, 1.49]

*Adjusted for when skill was introduced in intervention (see Table 2). Odds of reporting use was modeled within-person (days nested in participants)

The person- and day-level frequencies for each skill are presented in Table 4, along with the day-level odds of reporting use of each skill. Participants reported using willingness (44% of all days with surveys completed on time), time management (31% of days), and partner support (30% of days) most frequently, whereas engaging one’s values (17% of days) and effective goal-setting (3% of days) were used less frequently. Use of some skills changed over the course of the program (see Table 4); the odds of using (vs. not using) willingness and refocusing on a lapse (vs. relapse) increased by 11% each week in the program: (*OR* = 1.11, 95% *CI* [1.05, 1.18], *p* = 0.0003) and (*OR* = 1.11, 95% *CI* [1.05, 1.18], *p* = 0.0003), respectively. “Other” skills increased by 27% each week (*OR* = 1.27, 95% *CI* [1.14, 1.42], *p* < 0.0001). In contrast, the odds of using effective communication decreased by 12% each week (*OR* = 0.88, 95% *CI* [0.79, 0.97], *p* = 0.01). The day-level odds of using other skills did not meaningfully change over the course of the intervention program (see Table 4). Participants reported using an average of 1.82 skills per day (*SE* = 0.12, range = 0–8) and the number of skills used showed only 39% stability between-person. The number of skills used per day increased over time (*F*[1,2055] = 16.10, *p* < 0.0001, *sr* = 0.25), and were slightly lower on days with intervention sessions than days without (*F*[1,61] = 3.99, *p* = 0.05).

### Daily barriers to PA and associations with skills used

**Fig. 1:**
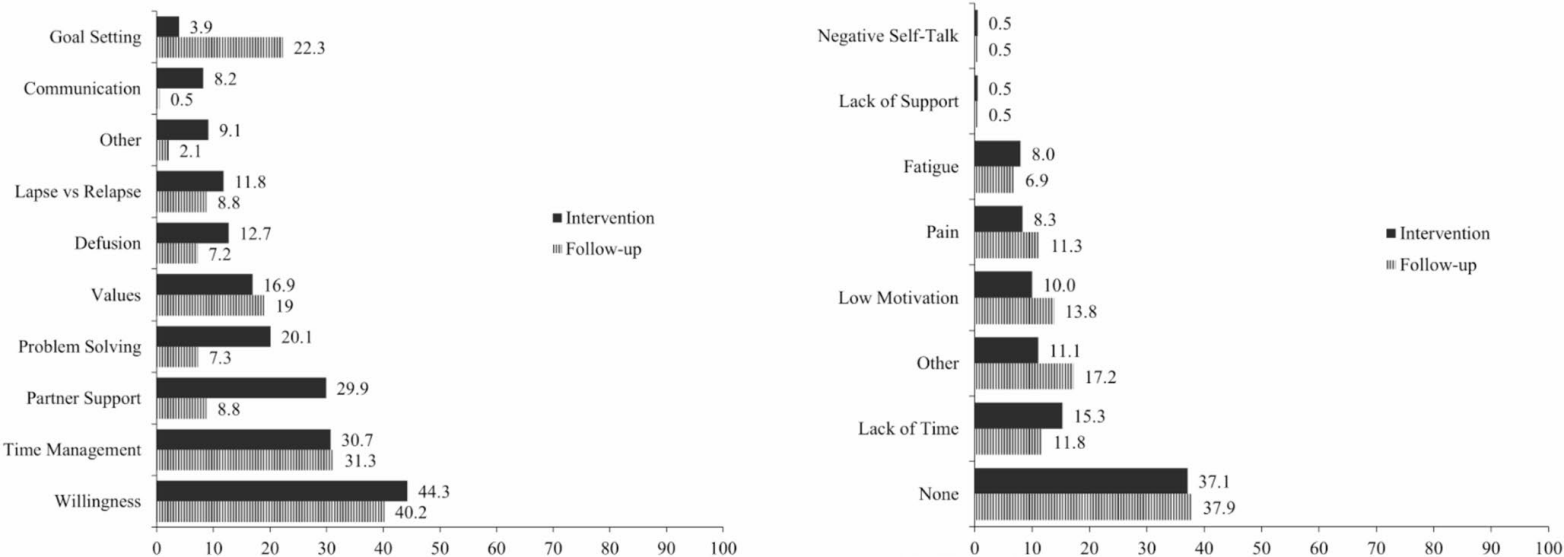
Day-level skills reported (% of all days), adjusted for when skill was introduced in intervention (panel A), and day-level barriers reported (% of all days, panel B)

As indicated, participants reported facing no barriers most frequently (37% of all days with surveys completed on time), followed by lack of time (15% of days), other barriers (11% of days), and low motivation (10% of days). The person- and sample-level frequencies for each barrier to PA are presented in Table 5. The stability of experiencing any barriers to PA (vs. not) was fairly low (*ICC* = 0.36), with 64% of the variability attributable to within-person fluctuation and error. The odds of experiencing any barriers was 0.64, 95% *CI* [0.55, 0.72], across the intervention and immediate post-intervention assessment periods, and the odds of experiencing any barriers (vs. not) decreased by 30% each week (*OR* = 0.70, 95% CI [0.60, 0.79], *F*[1,1913] = 4.64, *p* = 0.03) (Fig. 1).

**Table 5:** Daily physical activity barriers reported during the intervention and immediate post-intervention weeks of reporting

	Day level frequency n (%)	Person level M number of days [range]	Person level M% of days [range]
Fatigue	171 (8.0%)	2.8 [0–14]	8.6% [0–39.4%]
Lack of time	327 (15.3%)	5.2 [0–14]	17.3% [0–57.1%]
Lack of support	10 (0.5%)	0.2 [0–1]	0.5% [0–4.6%]
Low motivation	214 (10.0%)	3.4 [0–21]	12.0% [0–63.6%]
Pain	178 (8.3%)	2.9 [0–30]	9.1% [0–93.8%]
Negative self-talk*	4 (0.5%)	0.1 [0–2]	0.5% [0–9.1%]
Other	236 (11.0%)	3.8 [0–21]	12.7% [0–84.0%]
None	792 (37.1%)	12.8 [0–40]	39.7% [0–100%]

^*^Values adjusted for length of time barrier was introduced as a survey option

The odds of reporting barriers did not differ between days with and without intervention sessions (*OR* = 0.91, 95% CI [0.68, 1.22], *F*[1,1913] = 0.41, *p* = 0.52). However, the number of skills used was greater on days with no barriers reported than on days with barriers (*F*[1,59] = 188.84, *p* < 0.0001, *sr* = 0.47): participants used an additional skill on days without versus with barriers (*B*s = 2.23 vs. 1.41, respectively). CFA models showed that on days when barriers were reported, a type was observed such that participants reported use of no skills at all on 10% of those days. This pattern also emerged as a type on days with no barriers reported, though it was present for only 3% of those days. No notable anti-types, or patterns seen less often than chance, and no other notable types emerged on days with or without reported barriers.

### Skill use, daily barriers to PA, and PA behavior (steps per day)

**Fig. 2:**
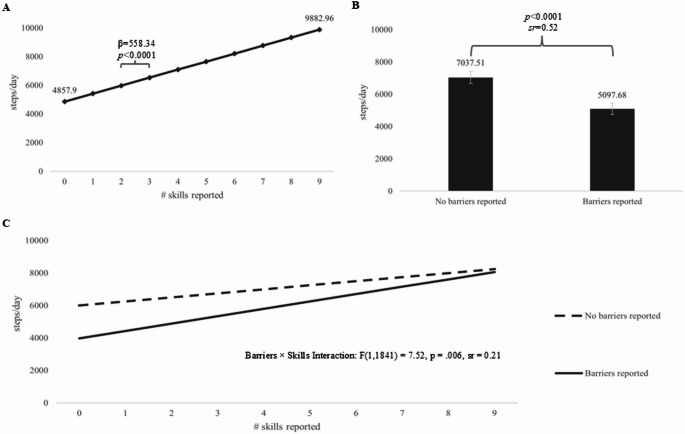
Steps per day by number of skills reported (panel A), if barriers were not or were reported (panel B), and the interaction of skills and barriers (panel C). Models control for week in program and between-person number of skills or total days with barriers reported, as appropriate

Neither the average number of skills used per day nor the overall number of days on which each participant reported experiencing a barrier to PA were related to their steps per day (between-person; *p*s > 0.08). However, controlling for week in the program and each participant’s average number of skills used per day, women engaged in ~ 550 more steps per day for each additional skill used (within-person; *F*[1,2051] = 180.85, *p* < 0.0001, *sr* = 0.47; see Fig. 2, panel A). Controlling for week in the program and each participant’s total number of days with barriers, they engaged in ~ 2000 more steps per day on days when they reported experiencing no barriers to PA (vs. days with a barrier – within-person; *F*[1,59] = 272.56, *p* < 0.0001, *sr* = 0.52; see Fig. 2, panel B). There was also a significant interaction between reporting barriers (yes/no) and the number of skills used (within-person; *F*[1,1841] = 7.52, *p* = 0.006, *sr* = 0.21): the positive association between skills used and steps per day was stronger on days with barriers, relative to days without barriers (see Fig. 2, panel C).

### Maintenance of skill use and barriers to PA at follow-up

For the 35 participants who completed the 3-month follow-up, end-of-day survey completion was 77% (188 completed on time/245 expected). The day-level frequencies of reporting each skill and barrier during the follow-up period are shown in Table 6. Similar to reports during the intervention and post-intervention assessment, willingness and time management were reported most frequently. In contrast, goal setting was also reported commonly at follow-up, which differed from reports during the intervention (22% of days at follow-up vs. 4% during the intervention). Problem solving, cognitive defusion, and communication were least frequent during the follow-up week. Reports of engaging partner support dropped from 30% of days during the intervention to 9% of days at follow-up. In addition, reports of barriers were similar at follow-up: participants reported facing no barriers most frequently, followed by other barriers (e.g., travel, weather, work responsibilities), low motivation, and lack of time.

**Table 6:** Skills and barriers reported during the 3-month follow-up assessment period (*n* = 35)

	Day level frequency *n* (%)	Person level *M* number of days [range]	Person level *M*% of days [range]
*Skills*			
Goal Setting	43 (22.3%)	1.2 [0–7]	22.5% [0–100%]
Time Management	61 (31.3%)	1.7 [0–6]	33.7% [0–100%]
Values	37 (19.0%)	1.0 [0–6]	17.4% [0–100%]
Willingness	80 (40.2%)	2.3 [0–7]	38.3% [0–100%]
Partner Support	17 (8.8%)	0.5 [0–5]	8.1% [0–100%]
Communication	1 (0.5%)	0.0 [0–1]	0.6% [0–20.0%]
Lapse vs. relapse (negative self-talk)	17 (8.8%)	0.5 [0–4]	8.2% [0–57.1%]
Problem Solving	14 (7.3%)	0.4 [0–4]	6.1% [0–57.1%]
Defusion	14 (7.2%)	0.4 [0–3]	9.0% [0–100%]
Other	4 (2.1%)	0.1 [0–1]	1.6% [0–14.3%]
*Barriers*			
Fatigue	12 (6.9%)	0.4 [0–3]	8.8% [0–100%]
Lack of Time	24 (11.8%)	0.7 [0–3]	10.5% [0–42.9%]
Lack of Support	1 (0.5%)	0.0 [0–1]	0.4% [0–14.3%]
Low Motivation	28 (13.8%)	0.8 [0–7]	13.2% [0–100%]
Pain	23 (11.3%)	0.6 [0–6]	11.9% [0–100%]
Negative Self-Talk	1 (0.5%)	0.0 [0–1]	0.5% [0–16.7%]
Other	35 (17.2%)	1.0 [0–6]	14.9% [0–85.7%]
None	77 (37.9%)	2.2 [0–7]	37.2% [0–100%]

## Discussion

The purpose of the present report was to describe the feasibility and acceptability of reporting on daily skill use during and after a PA skills training intervention, as well as the frequencies and co-occurrences of skill use with perceived barriers to PA and device-assessed PA behavior. We used an intensive (daily) assessment approach, for which participants were asked to reflect on their PA skill use and barriers to being active at the end of each day, with the goal of providing insight into behavior change skill use in daily life. Previous evidence in this area relied primarily on retrospective self-report, which is vulnerable to a range of recall biases (Smyth et al., 2017); as such, the present findings are likely to offer a more accurate and detailed understanding of skills training application between intervention sessions. We observed high compliance with the reporting protocol and participants’ overall ratings of acceptability were positive. Thus, the present findings contribute to a growing evidence base indicating that daily reporting on skill use is both feasible and acceptable among intervention participants (cf. Probst et al., 2018; Sala et al., 2023; Southward et al., 2022) and expands this finding to PA skills training.

During the present intervention and immediate post-intervention assessment, the PA skills used most frequently were willingness, time management, and PA partner support. Willingness (i.e., engaging in a valued behavior regardless of your immediate thoughts or feelings) may have resonated with participants because it aligned closely with our program philosophy, by promoting a more flexible (rather than a rigid, all-or-nothing) approach to PA. It is also likely that willingness was the most novel skill taught in this program, as it is less familiar to the general public than skills such as goal setting. Interestingly, the odds of using willingness increased over time, which may suggest that early success with it encouraged continued use. Alternatively, as this skill was introduced specifically to address low motivation, it may have become more relevant as motivation waned later in the program (cf. Knittle et al., 2018). Time management and partner support may have been particularly popular because they directly addressed some of the key overarching PA barriers identified by women in midlife (i.e., lack of time and insufficient social support for PA; McGuire, Seib & Anderson, 2016; McArthur et al., 2014)*.*

PA skills used least frequently during the present intervention were engaging values and goal setting. It is possible that values were perceived as too abstract (and thus, less tangible and more overwhelming than skills such as partner support) and women may need more training to use this skill effectively in daily life. Interestingly, although goal setting was integrated into the beginning and end of each PA skills session with coaches, it was not used frequently in daily life. The perceived importance and utility of frequent goal setting may have varied across participants, and some may have seen this as a weekly activity to do with their coach rather than something to do on their own each day. In addition, participants with negative past experiences related to goal setting may have more rigid ideas about this skill (i.e., all-or-nothing thinking). For example, behavior changes such as increasing exercise (e.g., in the context of weight loss efforts) are often associated with pressure or failure; women with these experiences may be less inclined to view goal setting as helpful (Deslippe et al., 2023; Casey, Civita, & Dasgupta, 2010; Kinnafick et al., 2018). It is also possible that participants did not differentiate goal setting from skills such as self-talk. As goal setting increased in use at 3-month follow-up assessment, however, frequent PA goal setting may be more useful during maintenance than during initial adoption efforts.

Beyond the frequencies of skill use, a valuable contribution of the current findings is our observation of considerable within-person *variability* in skill use. Skill use showed low stability for the same person across the intervention period, and this was due to linear increases or decreases for only a subset of skills. This suggests flexibility in participants’ application of skills and that skill use is likely adapted to meet the needs of shifting daily contexts. The number of skills participants used each day also increased (within-person) from the beginning to the end of the intervention, while PA barriers decreased, and participants perceived no barriers to PA on days when they used more (vs. fewer) skills. Perhaps most importantly, participants took more steps on days when they used more (vs. fewer) skills – particularly on days when they did (vs. did not) experience barriers to PA, with moderate to large effect sizes.

As a fundamental intention of many behavioral skills training programs is to provide participants with a diverse skill set from which they can select the skill that best meets their needs (Parsons et al., 2013), our findings indicate that this intention indeed manifests in daily life, between sessions. These findings also lend support to the notion that the same individual using more (vs. fewer) skills is associated with improvements in relevant outcomes (e.g., increases in state mindfulness, reductions in depressive or anxiety symptoms; Sala et al., 2023; Southward et al., 2022). Similarly, in psychotherapy research, participants whose skill application was more (vs. less) frequent and effective showed greater reductions in depression and anxiety and greater increases in life satisfaction, reflecting between-person associations (Fruhbauerova et al., 2024; Hundt et al., 2016; Jarrett et al., 2018; Terides et al., 2018). Moreover, daily reports showed that for the same participant, anxiety, stress, and suicidal ideation were lower on days following skill application (vs. no skill application), reflecting within-person associations (Probst et al., 2018; Sala et al., 2023; Southward et al., 2022). Participants also used more skills on days when they experienced greater-than-usual stress and anxiety (Southward et al., 2022), underscoring the importance of capturing day-level fluctuations in symptoms and related contextual factors.

Of note, an increase in the number of skills used per day may actually reflect inefficiency; for example, participants may have to try multiple skills before one “works.” This is a reasonable expectation for early stages of skills training, as participants practice using different skills to determine what works best for them. In later stages of training, however, it would be reasonable to expect that this practice makes trial-and-error less necessary, though participants also learn new skills that need practice. Additional work is needed to determine the efficiency, desirability, and effectiveness of using more versus fewer skills. In addition, previous research has called for assessing skill use *quality* (i.e., competency and effectiveness of using the skills taught to them), along with the frequency of skill use (Probst et al., 2018), which we did not do in the present study. Future research into the role of skill use quality will assist in providing a more holistic view of the role that skills play in PA promotion and maintenance efforts.

### Strengths, limitations, and additional future directions

Strengths of this study include the use of an intensive assessment design that included daily surveys and PA monitoring (via pedometers or personal PA tracking devices). This allowed for a more fine-grained assessment of PA skill use than is currently available, speaking to the translation of PA skills training to daily life between and following the end of structured intervention sessions. Skills were selected from evidence-based approaches to PA promotion (i.e., CBT and ACT) and were taught to participants by trained PA behavior change coaches. In addition, our use of a brief follow-up assessment provided initial insight into maintenance of PA skill use after the cessation of a formal program.

There were also several noteworthy limitations. The modest sample size limits statistical power for between-person tests, and the absence of a control or attention-matched comparison condition limits our ability to draw causal inferences about the effects of the intervention. In addition, we cannot determine the extent to which observed skill use in everyday life is attributable to skill acquisition via coaching or other features of the intervention. The present report includes a set of secondary analyses that were of interest from the design phase of the study, though many of the analyses were exploratory and did not include all tests of potential interest. For example, we did not test for associations between specific skills (or combinations of skills) used and PA behavior. Given that previous evidence shows that different skills may have different impact on outcomes (Hawley et al., 2017; Southward & Sauer-Zavala, 2022), such tests and their findings will be important for advancing this area of knowledge about behavioral interventions for PA. As in most PA programs, our coaches were able to introduce a skill and/or foreshadow a skill earlier than expected to support individualization of treatment. As daily surveys included all skills covered throughout the intervention and post-intervention period (Weeks 2–8), inconsistencies in time spent discussing and practicing a skill may have led to noise in our data.

In addition, some skills may have been taught to participants in other settings (e.g., psychotherapy) or encountered online or via self-help resources. Participants were also asked to recall skills used at the end of the day, and reports could be affected by how easy they were to recall and/or how much conscious effort was necessary to use the skill. Once skill use becomes habitual, it should not take much (if any) conscious effort to apply the skill (Haith & Krakauer, 2018), which could make it more difficult to recall and report. Future research using methods with even more temporal sensitivity (e.g., ecological momentary assessment) could determine when skills are used in real time. Our follow-up assessment was also optional and unplanned (i.e., added after some participants had already completed the program), potentially resulting in a self-selection bias from a subsample who benefited most from the program. Our inclusion of these results is for exploratory purposes and to support hypothesis generation, to be tested in future studies.

We also note that allowing the use of personal PA monitoring devices and self-reporting step totals from either a study-issued or personal device introduced imprecision in our estimates of PA behavior. However, as we focused on within-person patterns, small inaccuracies would have been consistent for each person across the intervention and thus, would not affect our results. Given that limited time and energy represent key barriers to PA for women in midlife, we believe that the downsides of this approach are outweighed by the convenience of using a familiar personal device (as available) and the self-reflection prompted by reporting step totals at the end of each day. Finally, although our intervention included a range of skills from evidence-based approaches to PA promotion, it was designed specifically for and tested with women in midlife who have elevated risk for CVD. Many participants in the present study also indicated high socioeconomic status and education level, which could influence both PA barriers and implementation of skills learned in the program: women in midlife with greater socioeconomic challenges may experience more substantial structural barriers to PA, and may need additional resources. It will be critical for future work to determine the generalizability of the present findings and identify subsets of skills that may work best for different groups, in addition to including a broader range of women to better capture this variation.

## Conclusions

In sum, intensive assessment of the use of PA skills in daily life during and after a PA promotion program can contribute to an improved understanding of day-to-day processes used to increase steps per day. Our findings underscore the feasibility and acceptability of this approach, which could ultimately lead to opportunities to refine PA promotion interventions (e.g., adjusting, replacing, or removing components that participants use less often in daily life). Additional work is needed to identify the specific mechanisms driving the translation of PA skills training to PA behavior change in daily life, among women in midlife and more broadly.

## Data Availability

Data and materials are available from the corresponding author upon reasonable request.
